# Virulence Factors for Hemolytic Uremic Syndrome, Denmark^1^

**DOI:** 10.3201/eid1005.030576

**Published:** 2004-05

**Authors:** Steen Ethelberg, Katharina E. P. Olsen, Flemming Scheutz, Charlotte Jensen, Peter Schiellerup, Jørgen Engberg, Andreas Munk Petersen, Bente Olesen, Peter Gerner-Smidt, Kåre Mølbak

**Affiliations:** * Statens Serum Institut, Copenhagen, Denmark; † The World Health Organization International Escherichia Centre, Copenhagen, Denmark

**Keywords:** Escherichia coli O157, Verocytotoxin, Stx Protein, Antibiotics, Cohort Analysis, Virulence Factor, Epidemiology, Serotype

## Abstract

We present an analysis of strain and patient factors associated with the development of bloody diarrhea and hemolytic uremic syndrome (HUS) among Shiga toxin-producing *Escherichia coli* (STEC) patients registered in Denmark in a 6-year period. Of 343 STEC patients, bloody diarrhea developed in 36.4% and HUS in 6.1%. In a multivariate logistic regression model, risk factors for bloody diarrhea were the *eae* and *stx*_2_ genes, O groups O157 and O103, and increasing age. Risk factors for HUS were presence of the *stx*_2_ (odds ratio [OR] 18.9) and *eae* (OR undefined) genes, being a child, and having bloody diarrhea. O group O157, although associated with HUS in a univariate analysis (OR 4.0), was not associated in the multivariate analysis (OR 1.1). This finding indicates that, rather than O group, the combined presence of the *eae* and *stx*_2_ genes is an important predictor of HUS.

Shiga toxin-producing *Escherichia coli* (STEC), alternatively known as Verocytotoxin–producing *E. coli*, is a major cause of gastroenteritis in developed countries (*1*). Symptoms range from mild to severe gastroenteritis with bloody diarrhea, and the infection may be complicated with hemolytic uremic syndrome (HUS) (*1*). STEC strains are defined by the Shiga (Stx) toxins; two classes, Stx1 and Stx2, are recognized (*2*). STEC strains also frequently harbor the locus of enterocyte effacement (LEE) pathogenicity island, which encodes intimin and a number of other virulence factors responsible for the intestinal attaching and effacing lesions (*2*). Several other factors may be involved in the pathogenic process, among them the enterohemolysin produced by many STEC strains (*2*). In several regions of the world, STEC strains of O group O157 are isolated from most patients, but STEC strains may belong to a large number of other O groups (*2*,*3*).

Only a few epidemiologic studies have addressed the relative importance of virulence factors for serious clinical disease. We studied a cohort of Danish STEC patients and determined risk factors for HUS and bloody diarrhea among a series of microbiologic and patient-related characteristics.

## Materials and Methods

### Patients and Isolates

From January 1, 1997, to May 1, 2003, 425 patients with STEC infections were registered in Denmark, a country of 5.4 million. These 425 registrations comprised the instances in which an STEC strain had been isolated from a fecal specimen obtained from a patient. In Denmark, clinical specimens for analysis for bacterial gastrointestinal pathogens are submitted to the Statens Serum Institut (SSI) or to 1 of 10 regional clinical microbiologic laboratories, depending on the county in which the physician who requests the analysis resides. In most patients (92%), infections were diagnosed from specimens sent to SSI, where the screening procedure was based on presence of the *stx* genes*.* Briefly, the diagnostic procedure consisted of isolating bacterial strains by using the SSI enteric media (*4*) and screening colonies with an *E. coli* morphology for *stx* genes by using colony hybridization with pooled DNA polynucleotide probes.

Subsequently, all isolates were referred to the national reference laboratory at SSI, and the STEC diagnosis was verified by using the Vero cell assay (*5*). The isolates were then further characterized for reference purposes. This process included full O:H serotyping (*6*) and determining the presence of the *stx*_1_, *stx*_2_, *eae* (intimin encoding), and *ehxA* (enterohemolysin encoding) virulence genes by hybridization to individual DNA polynucleotide probes.

### Patient Interviews

Starting January 1, 1997, STEC patients registered in Denmark have been routinely interviewed by physicians at the Department of Gastrointestinal Infections at SSI. This practice was initiated to identify possible outbreaks and to obtain data on the basic manifestations of disease and routes of infection. The patients (or their parents) were contacted by phone and interviewed by using a concise questionnaire. The questions concerned information on signs and symptoms, treatment, travel, contact with animals and other persons with similar symptoms, and whether the person, before becoming ill, had consumed items from a short list of foodstuffs and drinks that might potentially be associated with STEC infections. These interviews were the prime source of deciding whether the patients had HUS or bloody diarrhea, although for some patients, information from the requesting physicians was also obtained.

### Patient Data

A total of 82 patients were excluded from the cohort for various reasons. The isolates from 11 patients were not fully characterized with regard to virulence factors. For another 62 patients, details from interviews were insufficient, usually because patients could not be contacted by phone or because of language difficulties. Finally, two different STEC strains of different O groups were found in the fecal samples of nine patients. These strains were excluded because determining which isolate was responsible for the clinical symptoms was not possible. Among these nine patients were two with HUS. These were girls, ages l and 2 years, one of whom had bloody diarrhea. The girls were infected with isolates of O groups O145 and O156, and O groups O21 and O145, respectively. All four strains were *eae-*positive, *stx*_1_*-*negative, and *stx*_2_*-*positive.

Thus, the dataset used in the present analyses contained 343 patients. Nearly all of the illnesses were sporadic since no general outbreaks occurred in Denmark within the study period. A few patients may have been epidemiologically linked, however, by belonging to the same family or attending the same school or kindergarten.

The dataset was analyzed with respect to the two types of clinical outcomes, HUS and bloody diarrhea, by using a set of possible and available explanatory variables consisting of both microbiologic and patient factors. The microbiologic factors were O group and the absence or presence of each of the *eae*, *stx*_1_, *stx*_2_, and *ehxA* genes. By definition all isolates contained one or both of the *stx*_1_ and *stx*_2_ genes. The patient factors were age and gender; whether the patient had been hospitalized; diarrhea; bloody diarrhea (when analyzing for HUS); fever; vomiting; abdominal pain or joint pains; whether the patients had received antimobility agents or antimicrobial agents as a result of the infection; acquisition of the infection abroad; patient contact with other persons with similar symptoms or with animals (and if so which animals); and whether or not the patient, in the 5 days before symptoms developed, had eaten at a restaurant or consumed beef, lamb, pork, poultry, bean sprouts, unpasteurized milk, apple juice, or unwashed raw vegetables.

### Statistical Analyses

The statistical analyses were performed by using the SAS statistical package (Cary, NC). Variables were considered in a logistic regression analysis on the basis of statistical significance in a univariate analysis and biological relevance. Variables were subsequently eliminated from the logistic regression models if they were not significant at the 5% level or if confounding other explanatory variables and competing models were compared with likelihood ratio tests. Finally, the models were examined for statistical interactions; odds ratios (ORs) with 95% confidence intervals (CIs) were calculated.

All variables were dichotomous, except age. While the age distribution for the whole group of STEC patients and for patients with bloody diarrhea was only slightly skewed towards younger age groups, the HUS patients were young; of the 21 HUS patients, 18 were <8 years of age. For this reason, age was entered as an interval scale variable in the model for bloody diarrhea but as a dichotomous variable in the model for HUS, comparing the patients aged >8 years to those <7 years.

## Results

The cohort consisted of 343 patients; HUS had developed in 21 (6.1%), and bloody diarrhea had occurred in 125 (36.4%). The isolates comprised 74 serotypes and 49 different O groups. Among the O groups, O157 was the most common, comprising 81 (23.6%) isolates followed by O26 with 50 (14.6%) isolates and O103 with 45 (13.1%) isolates. A total of 28 different O groups occurred only once. Table 1 summarizes the O groups and the occurrence of HUS, bloody diarrhea, virulence genes, and median age of patients within the O groups. Of the patients, 32.2% had been hospitalized, 16.2% had received antibimicrobial treatment, and 16.4% had received treatment with antimobility agents as a result of the STEC infection; 22.0% reported having been infected on foreign travel.

**Table 1:** Number of isolates, clinical outcome, virulence factors, and median age for the O groups of STEC patients, Denmark^a^

O group	No. of isolates	HUS	Bloody diarrhea	*eae*-positive	*stx*_1_-positive	*stx*_2_-positive	*ehxA*-positive	Median age
O157	81	11	56	80	31	81	80	12
O26	50	3	16	50	42	12	45	1
O103	45	1	21	44	45	0	44	4
Other^b^	30	2	8	12	17	17	12	8
O rough	21	0	4	4	16	15	14	28
O117	19	0	1	0	19	0	1	32
O145	18	1	5	17	7	11	18	3
O146	18	0	4	0	13	15	11	48
O121	9	1	3	9	0	9	9	1
O91	7	0	0	0	6	5	4	25
O?^c^	7	0	0	1	4	3	3	25
O111	6	2	2	6	6	3	5	1
O128	6	0	0	0	6	5	5	31
O156	5	0	0	2	5	0	2	32
O55	4	0	1	2	4	0	0	3
O15	3	0	1	0	0	3	1	21
O70	3	0	0	3	2	1	3	1
O174	3	0	2	0	2	2	2	36
O5	2	0	1	1	2	0	2	30
O41	2	0	0	1	2	0	0	40
O76	2	0	0	0	2	0	1	42
O88	2	0	0	0	1	2	2	39
All	343	21	125	232	232	184	264	12

^a^STEC, Shiga toxin–producing *Escherichia coli*; HUS, hemolytic uremic syndrome. ^b^Includes all O groups that contained one isolate only. ^c^O?, O nontypeable.

### HUS

Among the 21 strains isolated from HUS patients, 11 were O group O157; 3 were O26; 2 were O111; and 1 each was O103, O121, O137, O145, and O165. Of the O157 strains, nine were O157:H7 and two O157:H-. All of the 21 strains were positive for the *eae* gene, while 18 strains were *stx*_2_-positive, 1 strain *stx*_1_-positive, and 2 strains *stx*_1_-and *stx*_2_-positive. Eighteen of the patients were <7 years of age. Table 2 shows the occurrence of bloody diarrhea, virulence genes, and median age of the patients within the O groups of the isolates from the 21 HUS patients.

**Table 2:** Number of isolates, instances of bloody diarrhea, virulence factors, and median age for O groups among 21 patients with hemolytic uremic syndrome

O group	No. of isolates	Bloody diarrhea	*eae-* positive	*stx*_1_-positive	*stx*_2_-positive	*ehxA-*positive	Median age
O157	11	8	11	0	11	10	4
O 26	3	2	3	0	3	2	1
O111	2	2	2	2	2	1	1
O103	1	1	1	1	0	1	1
O121	1	0	1	0	1	1	1
O137	1	0	1	0	1	1	3
O145	1	1	1	0	1	1	2
O165	1	1	1	0	1	0	0
All	21	15	21	3	20	17	1

ORs for each of the available possible determinants were calculated. On the basis of the possible determinants that were significantly associated with HUS within 90% confidence limits, a multivariate logistic regression model for HUS development was built. Because all strains from HUS case-patients contained the *eae* gene, the OR of this determinant could not be estimated, and the final model contained just three determinants: presence of the *stx*_2_ gene, bloody diarrhea, and age of <7 years. No statistical interaction was detected in the model. The details of the results of the univariate and multivariate analyses of the odds of developing HUS are shown in Table 3. Notably, O group O157, though significant in the univariate analysis, was not a risk factor. The OR for O157 declined from 4.0 (95% CI 1.6 to 9.7) to 1.1 (95% CI 0.4 to 3.1) when the other factors were included in the model. Of the other predictors, O group O111 was also associated with an increased risk for HUS in the univariate analysis but not in the multivariate analysis (Table 3).

**Table 3:** Risk factors for HUS among 343 STEC patients, Denmark, 1997–2003^a^

			Univariate analysis		Multivariate analysis	
Determinant	No. of patients	No. (%) with HUS	OR	95% CI	OR	95% CI
*eae*						
Negative	111	0	1			
Positive	232	21 (9.1)	ND^b^		NI	
*stx*_2_						
					
Negative	159	1 (0.6)	1		1	
Positive	184	20 (10.9)	19.3	2.6 to 145	18.9	2.4 to 146
Age						
>8 y	178	3 (1.7)	1		1	
<7 y	165	18 (10.9)	7.5	2.2 to 26.0	11.4	3.2 to 41.3
Bloody diarrhea						
No	218	6 (2.8)	1		1	
Yes	125	15 (12.0)	4.8	1.8 to 12.8	4.5	1.6 to 12.7
O157						
No	262	10 (3.8)	1			
Yes	81	11 (13.6)	4.0	1.6 to 9.7	NS	
O111						
No	337	19 (5.6)	1			
Yes	6	2 (33.3)	8.4	1.4 to 48.6	NS	

^a^STEC, Shiga toxin–producing *Escherichia coli;* HUS, hemolytic uremic syndrome; OR, odds ratio; CI, confidence intervals; NI, not included (test not appropriate); ND, not defined; NS, not statistically significant. ^b^p < 0.0002, Fisher exact test.

Eight of the HUS patients and 43 of the non-HUS patients had received antimicrobial treatment during the course of the STEC infection, and in fact the variable of being treated with antimicrobial agents as a result of the infection was also associated with an increased risk for HUS in a univariate analysis (OR 3.6; 95% CI 1.4 to 9.1) and when included in the multivariate analysis (OR 4.8; 95% CI 1.4 to 16.2). However, the antimicrobial treatment status was unknown for 29 of the non-HUS patients, so they were excluded from the analysis when the variable was included. The variable did not confound or interact with the three other determinants when added to the model.

The two remaining microbiologic traits, presence of the *stx*_1_ gene and the *ehxA* gene, were not associated with an increased risk for HUS in the univariate or multivariate analyses. Apart from the fact that all HUS patients had been hospitalized, the remaining available patient characteristics (i.e., sex, foreign travel, other symptoms apart from bloody diarrhea, contact with animals or other ill persons, and the food and drink exposures) were also not associated with increased risk for HUS.

### Bloody Diarrhea

An analysis was also made of risk factors for bloody diarrhea. On the basis of the patient interviews and the available clinical information, 125 patients with bloody diarrhea were identified. Of the strains isolated from these patients, 86% contained the *eae* gene and 67% the *stx*_2_ gene; 45% of the strains belonged to O group O157 and 17% to O group O103.

Again, ORs for each of the available putative determinants were calculated, and a logistic regression model was developed on the basis thereof. The final model contained the following five variables: *eae*-positive, *stx*_2_-positive, O group O157, O group O103, and age. Age was incorporated as an interval scale variable. The result of modeling indicated that all the above-mentioned variables were associated with an increased risk for bloody diarrhea (Table 4). Presence of the *eae* gene was the virulence factor that showed the closest association with development of bloody diarrhea, whereas the effect of presence of the *stx*_2_ gene was more modest. In contrast to what was found regarding HUS, O group O157 and O103 were independent risk factors for bloody diarrhea. In addition, there was statistical interaction (p = 0.03) between the determinants *eae*-positive and O group O103. However, only one O103 strain was *eae*-negative, and on this basis we choose to ignore this interaction.

**Table 4:** Risk factors for bloody diarrhea among 343 STEC patients, Denmark, 1997–2003^a^

			Univariate analysis		Multivariate analysis		
Determinant	No. of patients	No. (%) with BD	OR	95% CI	OR	95% CI	
*eae*							
Negative	111	17 (15.3)	1		1		
Positive	232	108 (46.6)	4.8	2.7 to 8.6	6.0	2.7 to 13.3	
*stx*_2_							
Negative	159	41 (25.8)	1		1		
Positive	184	84 (45.7)	2.4	1.5 to 3.8	2.5	1.2 to 5.1	
O157							
No	262	69 (26.3)	1		1		
Yes	81	56 (69.1)	6.3	3.6 to 10.8	2.7	1.2 to 5.7	
O103							
No	298	104 (34.9)	1		1		
Yes	45	21 (46.7)	1.6	0.9 to 3.1	2.8	1.2 to 6.3	
*ehxA*							
No	79	15 (19.0)	1				
Yes	264	110 (41.7)	3.0	1.7 to 5.6	NS		
Age^b^	-	-	-	-	1.3	1.2 to 1.5	

^a^STEC, Shiga toxin–producing *Escherichia coli;* BD, bloody diarrhea; OR, odds ratio; CI, confidence intervals; NS, not statistically significant. ^b^Calculated for an increase in age of 10 years.

Age was also found to be a risk factor for bloody diarrhea, but the risk increased with age as opposed to what was found for HUS patients (Table 4). In addition, there was an association (multivariate OR 2.2; 95% CI 1.1 to 5.0) between bloody diarrhea and indigenous infection (i.e., not infected while traveling abroad), although the status of this variable was unknown in 14 instances (data not shown). Finally, 29 of 117 bloody diarrhea patients and 22 of 197 nonbloody diarrhea patients had received antimicrobial treatment as a result of the STEC infection and, as for the analysis for HUS, the OR of antimicrobial treatment could be estimated after reducing the dataset by 29 cases. The univariate OR was 2.6 (95% CI 1.4 to 4.8), and upon inclusion of this variable in the logistic regression model, the multivariate OR was 3.0 (95% CI 1.4 to 6.3).

Virulence factors not associated with increased odds of bloody diarrhea included presence of the *stx*_1_ gene (not shown) and presence of the *ehxA* gene, although the latter, on the basis of the univariate analysis alone, was included as a risk factor (Table 4). The OR for presence of the *ehxA* gene was 0.9 (95% CI 0.4 to 1.9) when this variable was included in the model. None of the remaining patient characteristics was associated with bloody diarrhea in the multivariate analysis, although two symptoms were associated in the univariate analysis, namely fever (OR 1.8; 95% CI 1.1 to 2.9) and abdominal pains (OR 2.6; 95% CI 1.4 to 4.9). In addition, bloody diarrhea conferred an increased risk for hospitalization (OR 6.7; 95% CI 4.0 to 11.3).

## Discussion

This study presents an analysis of determinants of serious clinical disease among Danish STEC patients during a period of 6.33 years. The dataset we used stems from a full national cohort of largely sporadic cases. The strains have been collected and the patients interviewed prospectively. Furthermore, for >90% of the strains, the diagnostic screening methods have been based on presence of the *stx* genes, the very feature that distinguishes STEC from other *E. coli*. The dataset is therefore largely free of several types of otherwise common biases, such as those that may potentially arise through the process of selecting strains from strain collections, from studying outbreak strains, through missing patient information, or through surveillance directed towards certain serotypes such as O157:H7. For these reasons we find this dataset, although relatively small, to be well suited for an analysis of the impact of basic patient characteristics and virulence factors—in particular, the significance of different serotypes or O groups—on serious disease.

However, the limitations of the dataset should also be kept in mind. Among these is the relatively small number of available explanatory variables. At present, we have data on only a small series of virulence genes and no information on subtypes of individual genes. Also, the basic nature of the available patient information does not permit any type of detailed analysis of many patient-related factors that may play a role in the development of serious symptoms.

More than three quarters of the patients and roughly half of those complicated with HUS were infected with non-O157 STEC strains. The determinants associated with the development of HUS were the *stx*_2_ gene, the *eae* gene (indicating the presence of the LEE pathogenicity island), and young age. In addition, bloody diarrhea and treatment with antimicrobial agents were independently associated with HUS. O group O157 was not associated with an increased risk for HUS when we controlled for the presence of the *stx*_2_ gene. The apparent association with O157 seen in the univariate analysis was primarily due to the fact that all the O157 isolates contained the *stx*_2_ gene. Thus, our findings indicate that the potential of *E. coli* O157 strains to cause HUS may by and large be explained by the fact that these strains almost invariably encode Stx2 and the virulence factors of the LEE pathogenicity island.

The association between HUS and the *eae* gene, the *stx*_2_ gene, or both, has been observed in several other epidemiologic studies (*7*–*10*), although to our knowledge only one has been based on multivariate modeling (*11*). Although the approach taken in that study was somewhat different from ours, the conclusions were in line with ours; the authors found that, of a series of virulence genes including the *ehxA* gene, the *eae* and *stx*_2_ genes were the only significant determinants of HUS. Two other recent studies also point to the association between the *eae* and *stx*_2_ genes and the development of HUS. One compares virulence genes in non-O157 STEC strains from HUS patients with those from other infected persons in the United Kingdom (*12*); the other compares the frequency with which the two genes and the various O groups occur among German STEC patients with and without HUS (*13*).

In the analysis for bloody diarrhea, the determinants we found were, again, presence of the *eae* and *stx*_2_ genes, although the *stx*_2_ association was much less pronounced. In addition, the two most frequently occurring O groups, O157 and O103, were independent determinants. The latter finding indicates that additional virulence factors important for inducing bloody diarrhea may be present among isolates of these two O groups. Since bloody diarrhea was a risk factor in the analysis for HUS, this may also be of some importance for the development of HUS. We note that the single *stx*_2_-negative STEC strain isolated from an HUS patient was of O group O103.

In concordance with the findings of others (*2*), our results do not indicate that the *ehxA* gene plays an independent role in the pathogenic process leading to HUS or bloody diarrhea. The *ehxA* gene was not associated with HUS and although, on the basis of the univariate analysis, it appeared to be associated with bloody diarrhea, this association was no longer present in the multivariate analysis, i.e., after controlling for the determinants present in the model for bloody diarrhea.

Treatment with antimicrobial agents was a risk factor for both HUS and bloody diarrhea. This finding is of interest in light of the ongoing discussion of whether or not certain antimicrobial agents have the potential to induce serious clinical symptoms including HUS among patients with STEC infections (*14*). However, our data do not have sufficient clinical detail to determine the causal direction of this finding. Patients with HUS or bloody diarrhea initially have more severe symptoms than other STEC patients and may be more likely to receive antimicrobial treatment. Therefore, we may be observing a selection for more severe symptoms and not an effect of the antimicrobial agents per se.

Finally, we note that although only 9 patients out of the full cohort of 425 patients had two different STEC isolates simultaneously recovered from their stools, these 9 patients comprised 2 of the 23 HUS patients known to be present within the cohort. In both instances, both isolates were *eae-* and *stx*_2_-positive. This finding raises the possibility that different STEC isolates may complement each other in the pathogenic process leading to HUS and that such synergy may lead to an increased risk of developing HUS.

To conclude, our analyses indicate that the determinants associated with development of HUS are presence of the *eae* gene, presence of the *stx*_2_ gene, being a young child, and bloody diarrhea. Our analyses also indicate that the combined presence of the *eae* and *stx*_2_ genes is a better marker for the potential to cause HUS than is O group O157. This finding may have implications for the future planning of improved diagnostics and surveillance of STEC infections.
